# Supplementary material for: A paradigm shift in learning strategy research: Illustration and example of a within-person examination
Source: PLoS One. 2022 Sep 15;17(9):e0274548. doi: 10.1371/journal.pone.0274548 (PMC9477340; doi:10.1371/journal.pone.0274548)
Supplement: Attachment — Submitted filename: Response_to_Reviewers.docx [file pone.0274548.s002.docx]

In response to Reviewer #1

Thank you for reviewing my manuscript. I agree that the mathematical model for examining intra-individual variance has become complicated, and I tried to emphasize the importance of the intra-individual approach with plenty of descriptions and figures to complement it. I am honored that you appreciated that. I have prepared a response to your points below.

The writing is generally good, though technical. There are a few places where I think the translation to English has been uncertain. The most important is the use of "anyway" in labeling the study variables in contrast to "suitable". I understand the use of "suitable" but "anyway" doesn't fit as the opposite of "suitable." I would recommend the author review and reconsider tis label. More generally, a proofreading for English clarity by another, independent reader would be helpful.

- Thank you for pointing this out. First, I asked the proofreader to review and revise all the text in my manuscript to the extent that no changes were made to the content. Second, I have removed the expression "anyway" and replaced it with "irrelevant". With this change, the sentences about "anyway" has also been replaced with sentences about "irrelevant". Also, variables which used have been adjusted to use, so Cost_SA_ became Cost_SI_.

BEFORE: Notation of “anyway” and the sentences before and after it

AFTER: Notation of “irrelevant” and the sentences before and after it

Technically, the only concern I have is for the adequacy of the sample size to support the computation of so many parameters and then modeling them in multiple ways. It would be helpful for the author to address the degree of over or under determination in the it of the data to the multiple parameters.

- To estimate multiple parameters, the study employed a Markov chain Monte Carlo method, which passed the problem of the normality assumption for each parameter. However, as you pointed out, we did not discuss the necessary sample size. I have added a description to address this. In mixed-effects models, in addition to the sample size of participants, the number of items/stimuli that cross within participants is relevant to parameter estimation. Therefore, we discuss the number of participants and the number of items in terms of general sampling.

BEFORE: N/A

AFTER: page 19, line 697.

Similar to the sample size of items, the sample size of participants in this study is not large enough compared to other SRL studies. This study used MCMC methods to address multiple normality for estimating a large number of parameters, including variable effects with mixed effects models. On the other hand, the adoption of MCMC methods cannot be an exemption to small samples, and of course, large sample sizes are necessary for generalization of the study results.

In response to Reviewer #2

Thank you again for reviewing this paper. Your important remarks and numerous suggestions have helped me to improve it. I appreciate your statement that the consistency of the model proposed in this paper with other SRL studies and the position of this paper as an SRL study is now very clear. I have revised the paper based on your comments and suggestions.

Thank you for providing the Bayesian credible intervals for the L1 predictors. Am I reading this right: many of the CIs for costs of metacognitive strategies (SA, LA, SS) include 0. Does this mean that the costs of metacognitive strategies (other than SS) largely do not predict usage? If that is the correct interpretation, I would recommend emphasizing this important result more clearly in Study 2 and hypothesizing why more clearly in the discussion. The author compellingly explains why perceived costs often drive decision making. Why might the relationship between costs and use be near 0 when it comes to metacognitive strategies?

- The presentation of the Bayesian credible intervals makes the results of this paper clearer. Thank you for your suggestion. In the metacognitive strategies, variables that included 0 in the confidence interval (i.e., other than Cost_SS_) did not predict the degree of use for the learning strategy. As you pointed out, I did not explicitly state my interpretation of this result. I have added my interpretation below and would appreciate your review. Note that in Study 1 (Cognitive Strategies), as in Study 2, the results were not explicitly stated and there was no clear interpretation, so I have added them.

BEFORE: N/A

AFTER in Study 1: page 11, line 422.

Participants are more likely to associate cognitive strategies like memorization and comprehension with the study they are completing because these strategies are directly related to the acquisition of learning content. Therefore, a short time condition (e.g., the next test) and a condition in which participants did not consider the appropriate use of the cognitive strategy (e.g., just study for now) may have predicted the reduced use of the cognitive strategy. In other words, it is possible that the frequency of cognitive strategy use decreases only in the "short-term" and the "unconceivable situation" conditions in the subjective perceived cost held by the individual learner, but not in the other conditions.

AFTER in Study 2: page 15, line 527.

In Study 2, only Cost_SS_ predicted to Used, and the rest of Cost_SI_, Cost_LI_ and Cost_LS_ were determined not to predict strategy use because the 95% Bayesian credible interval contained 0. Metacognitive strategies have a role in controlling learning behavior rather than being involved in learning content. As with the cognitive strategies, the distant future condition does not predict the frequency of metacognitive strategy use, and it is possible that learners perceive studying as something to be done in the near future to show results on a test, both in terms of perceived benefit [25] and cost. It is interesting to note that unlike cognitive strategies, the conditions of use appropriate to the situation discouraged the use of metacognitive strategies. Metacognition, as the term implies, is a higher-order cognition of the target cognition/behavior, and thus may be difficult for participants to evaluate. Therefore, metacognitive strategies are difficult to use, and the results of this study (i.e., Cost_SS_ predicting Used, may reflect confusion about judging the "appropriate use" of metacognitive strategies in a situation.

On a similar note, although the strengths of the correlations were smaller for metacognitive strategies than cognitive strategies, I'd be hesitant to compare these correlations because different students responded to the cognitive and metacognitive surveys, I believe. The manuscript currently suggests that "Thus, for the variance among multiple strategies within a learner, the factors causing the variance differed between cognitive and metacognitive strategies." (line 547). I'd rewrite this sentence for clarity and also temper this language because I don't think that you have evidence to directly conclude that the factors that influence cognitive and metacognitive strategy use are different.

- Thank you for your suggestions and remarks. I agree that the comment here could not be supported by the results of the paper. I have made the following correction.

BEFORE: page 16, line 547.

Thus, for the variance among multiple strategies within a learner, the factors causing the variance differed between cognitive and metacognitive strategies.

AFTER: page 16, line 568.

Thus, it was shown that cognitive and metacognitive strategies may differ in their conditional knowledge of costs that lead to variance in the degree of use. However, because of the different participants and their sample sizes, the results of Studies 1 and 2 cannot be directly compared, and the differences in the behavior of within-individual variance between cognitive and metacognitive strategies should be mentioned with data measured on the same sample of these strategies.
