# Supplementary material for: A paradigm shift in learning strategy research: Illustration and example of a within-person examination
Source: PLoS One. 2022 Sep 15;17(9):e0274548. doi: 10.1371/journal.pone.0274548 (PMC9477340; doi:10.1371/journal.pone.0274548)
Supplement: Attachment — Submitted filename: Responses_to_Reviewers.docx [file pone.0274548.s003.docx]

Dr. Hannah Hausman

Guest Editor

*PLOS ONE*

Dear Dr. Hausman,

I would like to thank you for serving not only as a reviewer but also as a Guest Editor of my manuscript [PONE-D-21-02885, R1, and R2]. I am grateful for your advice to make my manuscript more readable. I have addressed each of your comments and suggestions as follows.

p. 19 line 709: Please use the term multivariate normality rather than multiple normality

RESPONSE: Thank you for pointing this out. I have corrected it.

BEFORE: p. 19 line 709

This study used MCMC methods to address multiple normality for estimating a large number of parameters, including variable effects with mixed effects models.

AFTER: p.21 line 748

The present studies used the MCMC method to address multivariate normality for estimating a large number of parameters, including variable effects with mixed-effects models.

Tables 1 and 4: Please use an abbreviated strategy names/label such as “Understand Relationships” rather than the cs4 etc.

RESPONSE: Thank you for your suggestion. I have labeled each of the abbreviated items. I have also associated this label with the strategy description in accordance with your next suggestion. In order to fit into the page layout, the table structure was varied: the means and standard deviations were combined in one cell, with the standard deviations in parentheses. The table title and note have also been partially changed accordingly. In the "Revised Manuscript with Track Changes" file, the old table was deleted because the serial numbers would shift if the old file was kept.

<Table1>

BEFORE: Label

cs1, cs2, cs3, cs4, cs5, cs6, cs7, cs8

AFTER: Label

Memorize Meaning, Understand Relationships, Memorize the Big Picture, Try to Understand, Ignore Origin, Ignore Meaning, Ignore the Big Picture, Just Write

BEFORE: Title

The mean (M) and standard deviation (SD) of each assessment item of cognitive strategies and the number of people with and without strategy knowledge in Study 1.

AFTER: Title

The mean and standard deviation (enclosed in parentheses) of each assessment item of cognitive strategies and the number of people with and without prior strategy knowledge in Study 1.

BEFORE: Note a

The cells in the "M" column show the number of people in "noticed (coded to 0.5)", and the cells in the "SD" column show the number of people in "did not notice (coded to -0.5)".

AFTER: Note a

The number at the top of the cell indicates the number of respondents who indicated they knew about the strategy (coded as 0.5), while the number at the bottom of the cell indicates the number of respondents who indicated they did not know about the strategy (coded as -0.5).

<Table 4>

BEFORE: Table 4

ms1. ms2, ms3, ms4, ms5, ms6, ms7

AFTER: Table 4

Predict Accomplishment, Clarify Understanding, Grasp Understanding, Evaluate Understanding, Study Planning, Appropriate Studying, Set Achievement

BEFORE: Title

The mean (M) and standard deviation (SD) of each assessment item of metacognitive strategies and the number of people with and without strategy knowledge in Study 2.

AFTER: Title

The mean and standard deviation (enclosed in parentheses) of each assessment item of metacognitive strategies and the number of people with and without prior strategy knowledge in Study 2.

BEFORE: Note a

The cells in the "M" column show the number of people in "noticed (coded to 0.5)", and the cells in the "SD" column show the number of people in "did not notice (coded to -0.5)".

AFTER: Note a

The number at the top of the cell denotes the number of respondents who answered indicated they knew about the strategy (coded as 0.5), while the number at the bottom of the cell indicates the number of respondents who indicated they did not know about the strategy (coded as -0.5).

I would recommend integrating the survey questions and responses into the text itself, rather than listing it at the end of the manuscript in the Supporting Information.

RESPONSE: Thank you for your suggestion. I have removed the "Supporting Information" segment and inserted those sentences into the "Measures" sections of Studies 1 and 2.

BEFORE: Supporting Information

AFTER: deleted

BEFORE: N/A

AFTER: p. 7 line 281

The items on cognitive strategy in Study 1 are as follows (as the survey instructions and questionnaire items are in Japanese [31] http://doi.org/10.15002/00010874, the author asked an English editing service editage) to perform back-translation to check the accuracy of the translation):

[Eight items]

The order in which the items are presented here corresponds to Table 1. Numbers in parentheses after the description indicate the items’ order of presentation in the questionnaire, whereas the labels in square brackets match the headings in Table 1. Questionnaires were randomly distributed in reverse order due to counterbalancing.

BEFORE: N/A

AFTER: p. 8 line 305

[New Paragraph]

Participants were required to respond to seven items for each strategy. The teaching and response items presented to the participants were as follows:

The following items indicate ways of studying that you may or may not use in your learning process. For each item, please circle a number for the following six: {1} How well does this apply to your current way of studying? {2} How cumbersome do you think it would be to use this method at all times, regardless of the circumstances, in order to improve your score on your next test? {3} How cumbersome do you think it would be to use this method only when it suits the situation in order to score points on your next test? {4} How cumbersome do you think it would be to use this method at all times, regardless of the circumstances, in continuing your future learning? {5} How cumbersome do you think it would be to use this method only when it suits the situation in continuing your future learning? {6} How effective do you think this method is? After “How well does this apply to your current way of studying?” for a given method, you will be asked {7} whether you “(A) were aware of this strategy beforehand” or “(B) were not aware about this strategy until reading about it on this questionnaire.” Please circle the corresponding letter. There are no right or wrong answers, so please answer based on what you think is accurate.

[Seven values]

BEFORE: N/A

AFTER: p. 15 line 516

The items of the metacognitive strategy in Study 2 are as follows:

[Seven items]

The order in which the items are presented here corresponds to Table 4. Numbers in parentheses after the description indicate the order of presentation in the questionnaire, whereas the labels in square brackets match the headings in Table 4. Questionnaires were also randomly distributed in reverse order due to counterbalancing.

The knowledge question asks participants to indicate if they were aware of the strategy. Throughout the manuscript, though, various terms such as “noticed” (p. 7 line 290) and “recognized” (p. 10 line 376) were used for this variable as well. These verbs don’t quite match the question. I would recommend use “knew of” or “were are of” instead.

RESPONSE: Thank you for pointing this out. I have modified these lines to improve their clarity.

BEFORE: p. 7 line 290

Knowledge regarding strategy was binary (“noticed” coded 0.5, or “did not notice” coded -0.5), and the other aspects were scored on a Likert scale from 1 (“Not at all”) to 6 (“Very true of me”).

AFTER: p. 9 line 326

Knowledge regarding strategy was binary (“knew of,” coded as 0.5, or “did not know of,” coded as -0.5), whereas the other aspects were scored on a Likert scale ranging from 1 (“Not true at all”) to 6 (“Very true”).

BEFORE: p. 10 line 376

First, knowledge regarding strategies (Knowledgeij)was coded “0.5” (for recognized) or “-0.5” (for did not recognize).

AFTER: p. 11 line 411

First, knowledge regarding strategies (Knowledgeij)was coded as 0.5 (knew about the strategy) or -0.5 (did not know about the strategy).

The cost questions specifically ask about whether using a strategy would be “troublesome” (pp. 19-20). “Cumbersome” (e.g., p. 14 line 402), “hard” (e.g., p. 16 line 563), and “difficult” (e.g., p. 16 line 536) were other words used to refer to costs. I know the survey questions were translated from Japanese for the manuscript, but I’m wondering if you could explain what the Japanese adjective for the cost questions would best translate to and what it would connote. Is it troublesome? Do cumbersome and hard work as synonyms as well? What I’m wondering is if the word that was used connotes anything about the strategy being logistically difficult (the way that the word cumbersome does) or is connoting something more along the lines of intellectually difficult (the way hard or difficult) might. In other words, were the cost questions likely having participants reflect on the practical effort of implementing a strategy (e.g., time, effort, resources) or was it more about the difficulty of the study experience itself (e.g., confusing, difficult, or tricky in the way that a challenging math problem can be). Clarifying the implied meaning of the Japanese adjective for that was used for “troublesome” in the cost survey questions could help readers understand which kinds of costs students generally avoid and could be relevant for designing future survey questions to tease apart different types of costs associated with a study strategy.

RESPONSE: Thank you for pointing out the lack of clarity in the wording. To quote your expression, I believe that "the practical effort of implementing a strategy (e.g., time, effort, resources)" is appropriate for my intended perceived cost of a learning strategy. The appropriate adjective placement for such costs was "intellectually difficult" for me, but the appropriate expression for this paper seems to be "cumbersome".

BEFORE: troublesome, hard, difficult

AFTER: cumbersome

Relatedly, the two suitable cost questions ask about using a strategy “only when it fits the situation.” However, there are places in the manuscript that mention whether the strategy is suitable for the content (e.g., abstract, p. 16 line 567). My understanding is that the actual survey questions didn’t ask about the suitability for the content, per se, but rather, the suitability for the situation in general? Is that accurate? Please clarify what the suitable questions were asking about. Also consider rephrasing “just study for now” (p. 11 line 425) accordingly.

RESPONSE: Thank you for your observation. I agree with your interpretation. This paper’s research design could only refer to the subjective situation of the learner.

BEFORE: Abstract

The analysis revealed that in terms of cognitive strategies (Study 1), learners avoided using learning strategies that they perceived as high cost to "use for the next test" and "keep using the strategy anyway, regardless of the content". On the other hand, in metacognitive strategies (Study 2), students avoided using learning strategies that they perceived as costly to "use for the next test" and "use as appropriate for the content".

AFTER: Abstract

The analysis revealed that, in terms of cognitive strategies (Study 1), learners avoided using learning strategies perceived to have a high cost in the short term; however, they used cognitive strategies regardless of the circumstances. Furthermore, regarding metacognitive strategies (Study 2), students avoided using learning strategies that they perceived as costly to use in the short term and used them only when they suited the circumstances.

BEFORE: p. 16 line 567

For cognitive strategies, the higher the subjective value of "it's hard to keep using it anyway for the next test," the less likely they were to use the strategy; On the other hand, metacognitive strategies showed the same correlation as cognitive strategies in short-term "when" conditional knowledge, such as "use it for the next test," but the higher the cost of "how" conditional knowledge, such as "use it according to the content," the less likely they were to use the strategy.

AFTER: p. 18 line 612

For cognitive strategies, the higher the subjective cost of a given strategy, the less likely participants were to use it;

Conversely, while metacognitive strategies showed the same correlation as cognitive strategies vis-à-vis temporal conditional knowledge (i.e., using a given strategy in the short term, such as in a future test), the higher the cost of a strategy’s methodology-related conditional knowledge (using a given strategy if the circumstances are suitable), the less likely were learners to use it.

BEFORE: p. 11 line 425

Therefore, a short time condition (e.g., the next test) and a condition in which participants did consider the appropriate use of the cognitive strategy (e.g., just study for now) may have predicted the reduced use of the cognitive strategy.

AFTER: p. 13 line 460

Therefore, a short timeframe (e.g., the next test) and a condition in which participants did not have to consider the appropriate use of the cognitive strategy (i.e., they did not take into account the circumstances) may have predicted the reduced use of the cognitive strategy.

In general, I would be careful not to use quotation marks when paraphrasing the question or interpreting the results unless you are directly quoting the (translated) survey question (e.g., abstract, p. 14 line 502, p. 16 line 562).

RESPONSE: Thank you for pointing this out. I have corrected them. I apologize for the inconvenience.

BEFORE: Abstract

The analysis revealed that in terms of cognitive strategies (Study 1), learners avoided using learning strategies that they perceived as high cost to "use for the next test" and "keep using the strategy anyway, regardless of the content". On the other hand, in metacognitive strategies (Study 2), students avoided using learning strategies that they perceived as costly to "use for the next test" and "use as appropriate for the content".

AFTER: Abstract

The analysis revealed that, in terms of cognitive strategies (Study 1), learners avoided using learning strategies perceived to have a high cost in the short term; however, they used cognitive strategies regardless of the circumstances. Furthermore, regarding metacognitive strategies (Study 2), students avoided using learning strategies that they perceived as costly to use in the short term and used them only when they suited the circumstances.

BEFORE: p. 14 line 502.

First, for the effect of the four perceived costs, CostSS, “is cumbersome to use properly for the next exam,” suppressed learning strategy use (gamma20 = -0.208 [-0.365–-0.038]).

AFTER: p. 15 line 551

First, for the effect of the four perceived costs, CostSS, “[this strategy] is cumbersome to use properly for the next exam,” suppressed learning strategy use (gamma20 = -0.208 [-0.365–-0.038]).

BEFORE: p. 16 line 562

For example, between-person correlations are interpreted as "the more troublesome the strategy is, the less likely it is to be used," while within-person correlations are interpreted as "the more troublesome a strategy is, the less likely it is to be used compared to other strategies that are not troublesome."

AFTER: p. 18 line 630

For example, between-person correlations are interpreted as "the more cumbersome the strategy is, the less likely it is to be used," while within-person correlations are interpreted as "the more cumbersome a strategy is, the less likely it is to be used, compared with less cumbersome strategies."
